# Diagnostic and therapeutic pitfalls in *NPM1*-mutated AML: notes from the field

**DOI:** 10.1038/s41375-021-01222-4

**Published:** 2021-04-20

**Authors:** Brunangelo Falini, Sofia Sciabolacci, Lorenza Falini, Lorenzo Brunetti, Maria Paola Martelli

**Affiliations:** Institute of Haematology, Centro Ricerche Emato-Oncologiche (CREO), Ospedale S. Maria della Misericordia, University of Perugia, Perugia, Italy

**Keywords:** Acute myeloid leukaemia, Cancer genetics

## Abstract

Mutations of *Nucleophosmin* (*NPM1*) are the most common genetic abnormalities in adult acute myeloid leukaemia (AML), accounting for about 30% of cases. *NPM1*-mutated AML has been recognized as distinct entity in the 2017 World Health Organization (WHO) classification of lympho-haematopoietic neoplasms. WHO criteria allow recognition of this leukaemia entity and its distinction from AML with myelodysplasia-related changes, AML with *BCR-ABL1* rearrangement and AML with *RUNX1* mutations. Nevertheless, controversial issues include the percentage of blasts required for the diagnosis of *NPM1*-mutated AML and whether cases of *NPM1*-mutated myelodysplasia and chronic myelomonocytic leukaemia do exist. Evaluation of *NPM1* and *FLT3* status represents a major pillar of the European LeukemiaNet (ELN) genetic-based risk stratification model. Moreover, *NPM1* mutations are particularly suitable for assessing measurable residual disease (MRD) since they are frequent, stable at relapse and do not drive clonal haematopoiesis. Ideally, combining monitoring of MRD with the ELN prognostication model can help to guide therapeutic decisions. Here, we provide examples of instructive cases of *NPM1*-mutated AML, in order to provide criteria for the appropriate diagnosis and therapy of this frequent leukaemia entity.

## Introduction

The *nucleophosmin* (*NPM1*) gene encodes for a multifunctional nucleolar protein with shuttling and chaperone properties [1, 2]. In 2005, we discovered that *NPM1* was mutated (often in association with *FLT3*) in about one-third of AML (mostly with normal cytogenetics) and that the NPM1 mutant protein was delocalized in the cytoplasm of leukaemic cells [3]. We subsequently proposed that *NPM1*-mutated AML represented a disease with distinctive clinical, pathological and molecular features [4] and promoted this concept within the Clinical Advisory Committees of World Health Organization (WHO). This eventually led to include *NPM1*-mutated AML as a new entity in the 2017 WHO classification of lympho-haematopoietic tumors [1, 5].

Clinical management of AML patients, including those with *NPM1*-mutated AML, is based upon the European LeukemiaNet (ELN) genetic-based risk stratification [6, 7]. *NPM1*-mutated AML without *FLT3*-ITD or with *FLT3*-ITD low allelic ratio (<0.5; *FLT3*-ITD^low^) belongs to the ELN favourable risk category whilst *NPM1*-mutated AML with *FLT3*-ITD high allelic ratio (≥0.5; *FLT3*-ITD^high^) falls into the intermediate risk group [6]. However, *NPM1*-mutated AML may carry a large variety of concomitant mutations that may influence its clinical course and prognosis [8, 9]. Thus, risk stratification of *NPM1*-mutated AML is an evolving area that in the future is expected to expand, including genotypes other than those currently recognized by the ELN. A recent study also points to the importance of race in risk stratification based on genomics [10].

Assessment of measurable residual disease (MRD) by real time quantitative polymerase chain reaction (RT-qPCR) for *NPM1* mutant transcripts [11] can be combined with the ELN risk stratification to inform therapeutic decisions, e.g. helping to select patients who may benefit from allogeneic haematopoietic stem cell transplantation (allo-HSCT) [7]. *NPM1* mutations are an ideal target for monitoring MRD since they are AML specific [12], common [3], stable and do not drive clonal haematopoiesis [13]. Moreover, *NPM1* mutant transcripts are expressed at high levels, allowing sensitivity up to 1:10^5^–1:10^6^.

Despite most patients achieving MRD-negativity may still have up to 10^7^ residual leukaemic cells, they do not relapse, suggesting that the host immune system may control or eradicate the residual disease. Interestingly, T lymphocytes reactive against HLA-presented NPM1 mutant neoantigens were demonstrated in patients [14]. Conversely, about 30% of MRD-negative cases relapse [9], probably because their immune system is unable to clear the residual leukaemic cells, especially when *FLT3*-ITD (imparting a high proliferative index) is present.

Whatever is the mechanism of MRD eradication, achieving MRD negativity in PB [9] or BM [15] or a marked drop of MRD in BM [16, 17] or in PB [18] is predictive of low risk of leukaemia relapse and good survival. Pre-allotransplant monitoring of MRD is also important for predicting outcome, since MRD-positivity in this setting is associated with poor outcome [19–21]. In fact, although allo-HSCT is effective in eradicating MRD, post-transplant MRD negativity is more durable in patients who are MRD-negative before allo-HSCT [22]. Nevertheless, myeloablative allo-HSCT [20] still performs better than standard CHT [18, 23].

Nearly 50% of MRD-positive patients with <1000–2000 *NPM1* mutant transcripts copies/10^5^ *ABL* [24] at the end of CHT achieve MRD negativity spontaneously or retain stable low-level expression without relapsing at a minimum follow-up of 8 months [25]. Patients with both *FLT3*-ITD and <4 log reduction in *NPM1* transcript levels at the end of CHT are at high risk of disease progression and should be considered for pre-emptive treatment [25].

In the everyday clinical practice, haematologists involved in management of AML patients have sometimes to face with diagnostically complex cases and difficult therapeutic choices. Here, we present 6 challenging *NPM1*-mutated AML cases, in order to provide criteria for the appropriate diagnosis and therapy of this common leukaemia entity.

## Case 1: adult young patient with *NPM1*-mutated AML, multilineage dysplasia and clonal evolution of *FLT3*-ITD

**Fig. 1:**
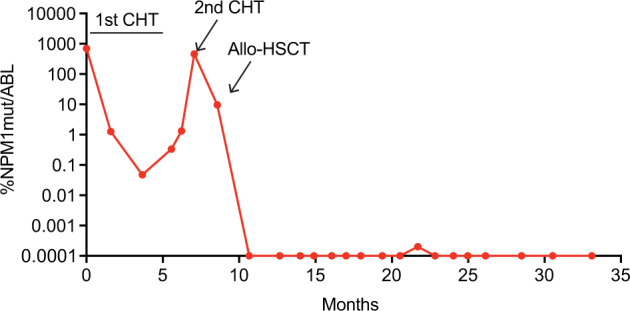
*NPM1* MRD monitoring by RT-qPCR in case 1. Monitoring of *NPM1*mut transcripts during therapy and follow-up (see text). 0.0001%NPM1mut/ABL is equivalent to MRD negativity. 1st CHT, first line chemotherapy; 2nd CHT, salvage chemotherapy; allo-HSCT, allogeneic haematopoietic stem cell transplant.

A 58-year-old woman presented with urinary tract infection. The complete blood count (CBC) showed: white blood cells (WBC) 12.8 × 10^9^/L, haemoglobin (Hb) 9.5 g/dL and platelets 144 × 10^9^/L. The bone marrow (BM) showed AML with multilineage dysplasia (MLD). *NPM1* mutation A and *FLT3* wild-type were detected. The BM karyotype was normal. She was treated with a ‘7 + 3’ regimen, achieving a complete remission (CR) and 2.7 log reduction of *NPM1* mutant transcripts at RT-qPCR (Fig. 1). The patient then received two idarubicin/cytarabine-based consolidation cycles. In the following months, we observed a progressive increase of *NPM1* MRD in the BM (Fig. 1) that was rapidly followed by haematological relapse (15% blasts). A small *FLT3*-ITD subclone (0.5%) had also appeared. She received a combination of fludarabine, cytarabine, idarubicin and etoposide that led to CR and 1.7-log reduction of *NPM1* transcripts (Fig. 1). Allo-HSCT from an haploidentical donor was performed and she is now in molecular CR, almost 2 years after allotransplant (Fig. 1).

### Questions and recommendations

**Fig. 2:**
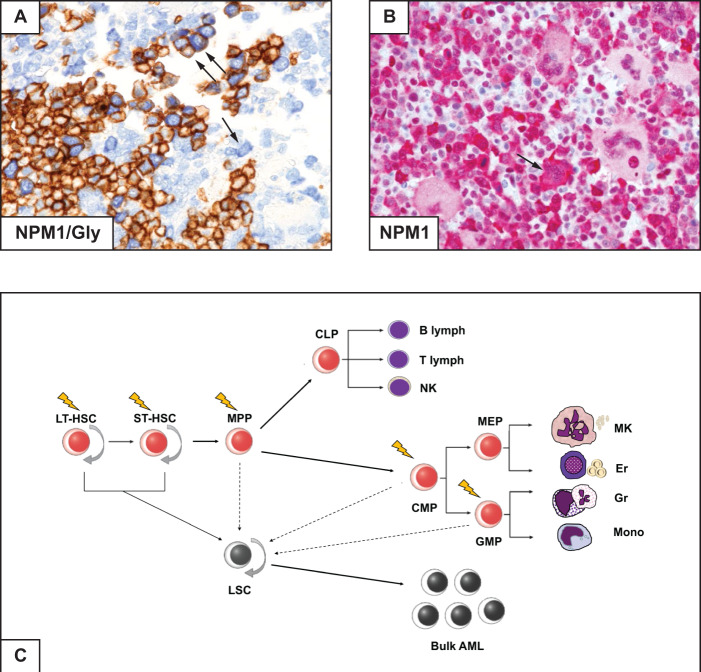
AML with multilineage dysplasia and cell of origin of *NPM1*-mutated AML. **A** Multilineage involvement is documented by the presence of myeloid blasts (single arrow) expressing cytoplasmic NPM1 (blue) and immature erythroid cells (double arrows) expressing cytoplasmic NPM1 (blue) and surface glycophorin (brown) (double staining for NPM1/immune-alkaline phosphatase APAAP technique and glycophorin/ immunoperoxidase, x400). **B** Another area of BM biopsy showing cytoplasmic NPM1 in dysplastic megakaryocytes (arrow) and myeloid cells (APAAP immunostaining; x400) **C** Cartoon depicting the putative cell of origin of *NPM1*-mutated AML. Lighting bolts indicate the suggested putative cells of origin.

Our patient showed AML with MLD (i.e. dysplasia in at least 50% of cells, in at least two BM cell lines [5]) and expression of cytoplasmic NPM1 in different haematopoietic cell lineages (Fig. 2A, B). MLD is one of the diagnostic criteria defining AML with myelodysplasia related changes (AML-MRC) but, according to WHO classification, when it coexists with *NPM1* mutation, the genetic lesion supersedes morphology and the case should be diagnosed as *NPM1*-mutated AML [5, 7, 26]. Conversely, a previous history of myelodysplastic syndrome (MDS) or MRC-related cytogenetic abnormalities, even in the presence of *NPM1* mutation, are diagnostic of AML-MRC [5]. Thus, our patient was diagnosed as *NPM1*-mutated AML since only MLD but no history of MDS or MRC-related cytogenetic abnormalities were documented.

MLD also helps defining the cell of origin of *NPM1*-mutated AML. Studies in mice suggest that *NPM1*-mutated AML may derive from a granulocytic-monocytic progenitor (GMP) [27]. We propose it may also originate from either a common myeloid progenitor (CMP) or a haematopoietic stem cell (HSC) (Fig. 2C), for the following reasons: (i) Erythroid and megakaryocytic cell lineages frequently show cytoplasmic NPM1 (Fig. 2A, B) or mutations in microdissected cells [28]; (ii) Precursors carrying *NPM1* mutations with the immunophenotype of leukaemic stem cells (CD34 + /CD38-/CD123 + /CD33 + /CD90-) are present in BM of *NPM1*-mutated AML patients [29]; and (iii) Human *NPM1*-mutated CD34 + AML cells transplanted into NSG mice generate an AML recapitulating the original patient’s disease, with monocytic differentiation and loss of CD34 [29].

Because our patient belonged to the ELN favorable risk category (*NPM1*-mutated without *FLT3*-ITD) [6], she received induction plus consolidation without allo-HSCT. In fact, patients with this genotype have <40% cumulative risk of relapse and high probability to achieve a second CR and to be salvaged by allo-HSCT. Allo-HSCT in first CR (CR1) has been proposed for patients <50 years, with low transplant-related risk and HLA-identical donor [30], but this option remains investigational [7]. Thus, we only administered consolidation CHT.

MRD monitoring documented an early molecular relapse [24] shortly followed by haematological relapse. The molecular relapse may have been heralded by the MRD suboptimal reduction after two cycles of CHT [9]. This could serve as another reasonable criterion for favoring allo-HSCT in CR1 [9] in otherwise favorable risk *NPM1*-mutated AML according to ELN [6]. Currently, this issue is addressed in a multicenter, MRD-driven study on AML patients with favorable/intermediate-risk sponsored by Gruppo Italiano Malattie EMatologiche dell’Adulto, GIMEMA (NCT04168502). Sequential monitoring for MRD may allow pre-emptive intervention before haematological relapse [31–33].

The emergence of a small *FLT3*-ITD subclone (0.5%) at relapse suggests clonal evolution towards a more aggressive AML. *FLT3*-ITD is an unstable mutation that may be lost (if present at diagnosis) or acquired at relapse. Interestingly, 4/6 *NPM1*-mutated AML patients with *FLT3* wild-type at diagnosis who relapsed with *FLT3*-ITD (according to conventional PCR), harbored very small *FLT3*-ITD subclones already at diagnosis, when analyzed by a highly sensitive patient-specific RT-qPCR for *FLT3*-ITD [34].

Therefore, the patient was treated with salvage CHT followed by allo-HSCT. She did not receive an FLT3 inhibitor since it was not approved at that time. Salvage CHT induced a new CR and about 2-log reduction in *NPM1* transcripts. Although *NPM1* MRD positivity before allo-HSCT has been associated with higher relapse rate [19–21], our patient is still in molecular CR almost 2 years after allotransplant.

## Case 2. Older fit patient with *NPM1/FLT3-*TKD*/DNMT3A* triple mutated AML, trisomy 8 and extramedullary disease

**Fig. 3:**
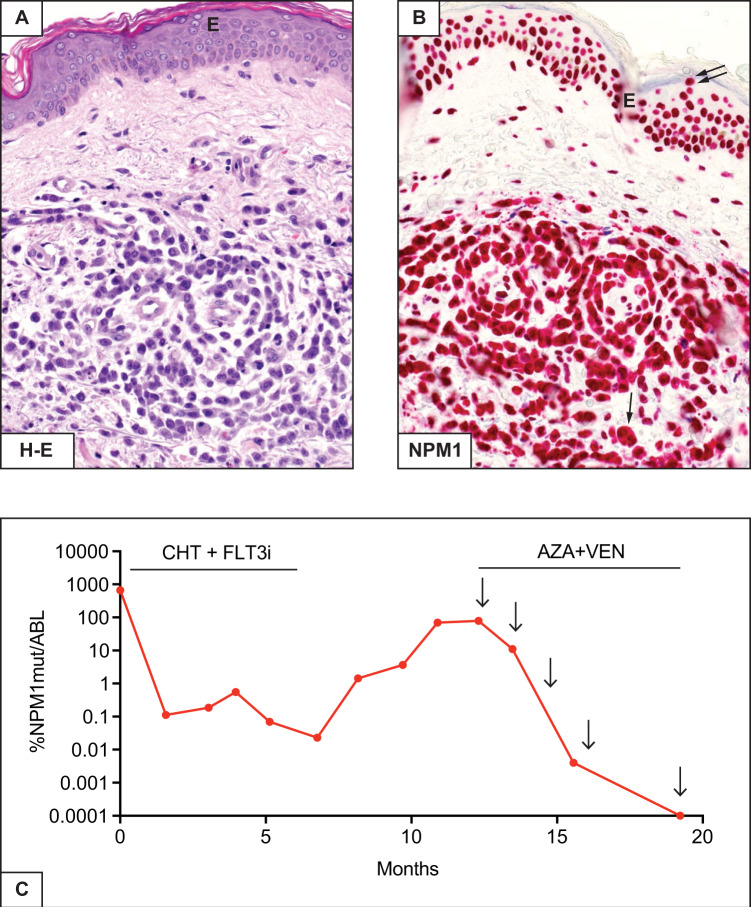
*NPM1/FLT3*-D835/*DNMT3A* triple-mutated AML with skin involvement. **A** Skin biopsy showing marked dermal infiltration by leukaemic cells. E indicates the overlying epidermis (Haematoxylin-Eosin, x400). **B** Leukaemic cells express cytoplasmic NPM1 (single arrow) whilst the cells of the overlying epidermis show nucleus-restricted NPM1 positivity (double arrows) (APAAP immunostaining, x400). E indicates the overlying epidermis. **C** MRD monitoring by RT-qPCR. 0.0001%NPM1mut/ABL is equivalent to MRD negativity. CHT + FLT3i, chemotherapy plus midostaurin; AZA + VEN, azacytidine plus venetoclax. Arrows indicate the beginning of each cycle of azacytidine plus venetoclax.

A 72-year-old woman presented because routine examinations revealed WBC 35.8 × 10^9^/L, Hb 12.6 g/dL and platelets 84 × 10^9^/L. The BM was diffusely infiltrated by myelomonocytic leukaemic cells expressing cytoplasmic NPM1. Mutations of *NPM1* (type A), *FLT3*-D835 and *DNMT3A* were detected. The BM karyotype revealed a trisomy 8 in about 20% of metaphases. Multiple light-purple skin nodules were found on clinical examination and a skin biopsy revealed dermal infiltration by leukaemic cells with cytoplasmic NPM1 (Fig. 3A, B). She received a ‘7 + 3’ regimen plus midostaurin, achieving haematological CR. Disappearance of *FLT3-*D835 mutation and a 3.8-log reduction of *NPM1* mutant transcripts were observed (Fig. 3C). The patient received two cytarabine-based consolidation cycles plus a FLT3 inhibitor (midostaurin) achieving a maximum of 4.5-log reduction of *NPM1* MRD (Fig. 3C); *FLT3*-D835 remained negative. In the following months, *NPM1* MRD in the BM progressively increased (Fig. 3C), the patient remaining in haematological CR. No skin lesions were observed. She received pre-emptive therapy with venetoclax plus the hypomethylating agent (HMA) 5-azacytidine. She is now in molecular CR at the fifth cycle of this combo (Fig. 3C).

### Questions and recommendations

Our patient carried the D835 mutation involving the tyrosine kinase domain (TKD) of *FLT3*. Accordingly, we administered CHT plus midostaurin. The benefit of using this combo for the *NPM1*-mutated/*FLT3*-TKD genotype was shown in a recent study [35]. However, the best post-remission therapy for these patients (consolidation CHT vs allo-HSCT) remains controversial. In fact, *FLT3*-TKD mutations have been associated with favorable prognosis in some studies [36–38] but not in others [39, 40]. The *NPM1*-mutated/*FLT3*-TKD genotype in the RATIFY trial showed a 5-year OS rate of 70% [35]. Thus, patients receiving CHT plus midostaurin should be probably not allotransplanted in CR1 and we adopt this strategy.

Then, the question raises whether the early molecular relapse in our patient could have been related to the concomitant *DNMT3A* mutation. In fact, *NPM1*-mutated AML elderly patients co-mutated for *DNMT3A* seem to have high risk of relapse even though they achieve good molecular responses post-induction CHT. They should be considered for allo-HSCT or maintenance strategies, even if they belong to the favorable ELN risk group [41].

*NPM1*-mutated AML co-mutated for *FLT3*-ITD and *DNMT3A* was initially recognized to display distinctive microRNA and epigenetic features [42]. The triple-mutated AML^*NPM1/FLT3-*ITD*/DNMT3A*^ is clinically characterized by high WBC count, monocytic phenotype, absence of multilineage dysplasia, extramedullary involvement, normal karyotype and poor outcome [8, 43, 44]. Our triple-mutated patient carried *FLT3-*TKD instead of *FLT3*-ITD. Although it is difficult to make a comparison based upon only one case, she exhibited clinical and biological features similar to those of typical AML^*NPM1/FLT3-*ITD*/DNMT3A*^ and responded poorly to CHT plus midostaurin. The introduction of more powerful FLT3 inhibitors may increase the percentage of triple-mutated patients who may be bridged to allo-HSCT. Preclinical studies suggest that triple mutated AML^*NPM1/FLT3-*ITD*/DNMT3A*^ may be sensitive to venetoclax [45]. Our patient responded to pre-emptive therapy with venetoclax plus HMA, but this observation requires further clinical validation.

Did the trisomy 8 conditioned the suboptimal response to therapy in our case? About 15% of *NPM1*-mutated AML exhibit an abnormal karyotype [3], usually characterized by +8, +4, -Y, del(9q) and +21, monosomy of chromosomes 5 and 7 and complex karyotype being very rare [46]. These aberrations represent secondary late events during the clonal evolution of *NPM1*-mutated AML [46]. Thus, trisomy 8 (as in our case) has no prognostic impact in *NPM1*-mutated AML [46]. Adverse-risk aberrations in *NPM1*-mutated AML include monosomy 7 or 5 or *TP53* deletion [47]. However, cases with these genetic abnormalities would fill in the WHO category of AML-MRC.

Skin lesions, as in our patient, have been described in association with *NPM1*-mutated AML showing monocytic features [48]. Whether skin involvement represents a poor prognostic factor in the context of our patient’s genotype remains unknown. In a study including >3,000 AML patients, extramedullary disease did not show an independent prognostic value [49] but mutational landscape was not investigated. Interestingly, our patient relapsed in the BM but not in the skin.

## Case 3. Older unfit patient with *NPM1*-mutated AML without *FLT3*-ITD presenting during the COVID-19 pandemic

An asymptomatic 74-year-old woman presented during the COVID-19 pandemic with a routine CBC revealing WBC 2.1 × 10^9^/L, Hb 7.6 g/dL and platelets 109 × 10^9^/L. BM examination showed infiltration by myelomonocytic leukaemic cells that were negative for CD34, positive for myeloperoxidase and macrophage-restricted CD68, and expressed cytoplasmic NPM1. The *NPM1* mutation A was detected whilst *FLT3* was wild-type. The BM karyotype was normal. The patient, considered unfit for CHT, received venetoclax plus 5-azacytidine. She achieved MRD-positive CR after the first cycle and is now in CR MRD-negative after five cycles of this combo.

### Questions and recommendations

What is the best treatment available for our patient? Venetoclax plus HMA or low dose cytarabine (LDAC), induced CR in 70–90% of *NPM1*-mutated AML patients [50, 51]. Similar dramatic responses have been also reported in *NPM1*-mutated myeloid sarcoma [52, 53]. Thus, the standard therapy for older unfit *NPM1*-mutated AML patients is now venetoclax plus HMA [7, 50]. CR is frequently achieved after one cycle and the regimen is usually well tolerated, with an early mortality of only 7% [51]. Nevertheless, patients experience a prolonged drug-related pancytopenia that should be differentiated from refractoriness to therapy by BM evaluation at appropriate time points. Persistent neutropenia may require prolonging intervals between cycles, reducing the duration of venetoclax administration per cycle or using granulocyte colony-stimulating factor.

Venetoclax-based regimens have been also recommended as temporary alternative to intensive CHT in older fit *NPM1*-mutated AML patients during COVID-19 pandemic [54, 55]. MRD assessment may be particularly helpful under these circumstances, although the optimal time points for monitoring need to be established for this combo [56].

Venetoclax plus HMA is also approved by the Italian Drug Agency as frontline therapy for fit AML patients ≥75 years old. In *NPM1*-mutated AML patients >65 years old, this combo compared favorably with intensive CHT [57]. However, these results need to be confirmed in randomized prospective studies. We currently treat fit *NPM1*-mutated AML patients >60 but <75 years old with standard CHT, although outcome remains poor, independently of *FLT3* status (3-year overall survival of 35%) [58]. Older eligible patients, who achieve complete remission after one or two cycles of CHT, should be offered reduced-intensity conditioning [59] or non-myeloablative [60] allo-HSCT.

## Case 4. Pediatric *NPM1*-mutated AML patient with unusual co-mutations

A 17-years-old girl presented with fever, cough and abdominal pain. The CBC showed WBC 1.3 × 10^9^/L, Hb 8.0 g/dL and platelet 25 × 10^9^/L. The BM was diffusely infiltrated by monoblasts positive for macrophage-restricted CD68 and negative for myeloperoxidase and CD34. Next generation sequencing (NGS) of 43 genes detected a previously unnoted *NPM1* mutation (see below), a rare *FLT3* N841H mutation and two *TET2* mutations. The BM karyotype showed a trisomy 8 in 4/25 metaphases. She received a ‘7 + 3’ induction, achieving CR. NGS after the first consolidation cycle showed disappearance of *NPM1* mutation but persistence of *FLT3-*N841H. Parents asked for a consultation since allo-HSCT from the HLA-identical sister was proposed at another Institution.

### Questions and recommendations

*NPM1* mutations are much less frequent in the childhood than in adults (about 7% vs 30%) [61]. Such a difference could be related to the fact that, in order to occur, *NPM1* mutations require a background of clonal haematopoiesis (usually driven by *DNMT3A* and *TET2* mutations) that is a very rare event in children but progressively increases with age [62]. NGS revealed a new *NPM1* mutation (deletion of two nucleotides and an insertion of four nucleotides at position 864) leading to loss of tryptophans 288 and 290 and the creation of a putative nuclear export signal (NES) at the NPM1 C-terminus, both required for cytoplasmic dislocation of NPM1 [63]. Given the rarity of this *NPM1* mutation, NGS and patient-specific RT-qPCR represent the best methods for MRD monitoring in this case.

What is the significance of the accompanying *FLT3* and *TET2* mutations in our patient? The *FLT3*-N841H mutation is located in the amino-terminal portion of TKD (activation loop of *FLT3*) and had previously reported only in one old patient with de novo AML FAB-M5 [64]. *FLT3*-N841H causes substitution of histidine for asparagine at codon 841 and leads to conformational changes of activation loop similar to substitutions of histidine for isoleucine (N841I) or tyrosine (N841Y) [65] that were previously shown to have transforming properties. Notably, NGS analysis of BM sample in CR after the first consolidation cycle showed disappearance of *NPM1* mutation but persistence of *FLT3*-N841H at 49% VAF. This finding raised the question whether the mutation was germline or related to clonal haematopoiesis [66]. Presence of *FLT3*-N841H in patient’s hair demonstrated its germline origin. The two mutations of *TET2* were missense and most likely not pathogenetically relevant. The lack of impact of trisomy 8 in risk stratification of *NPM1*-mutated AML patients has been already discussed in case 2.

What is the best post-remission treatment for this young girl? Although the ELN stratification model [6] mainly applies to adult patients age 18–60 years, *NPM1* mutations appear to be a prognostic predictor even in children [67]. Because the *FLT3*-N841H mutation is germline and not functionally relevant, our patient should be assigned to the ELN favorable risk and receive only CHT without FLT3 inhibitors. No allo-HSCT in CR1 is recommended either.

## Case 5: AML with *NPM1* exon 11 mutation

**Fig. 4:**
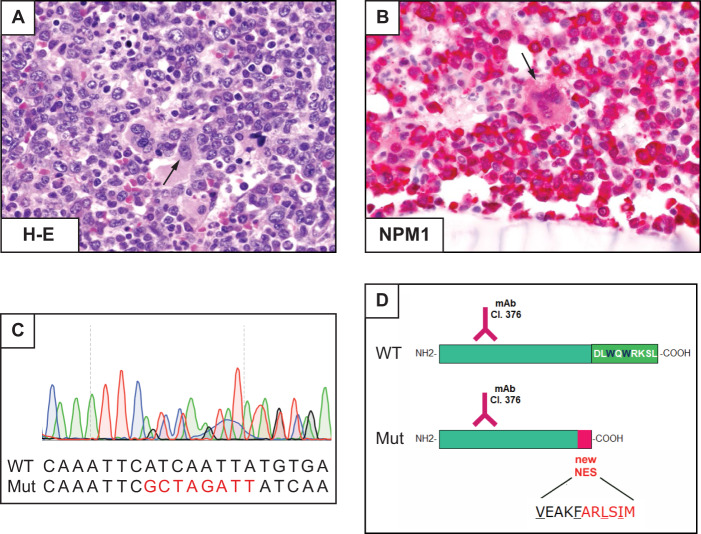
AML with mutation of *NPM1*, exon 11. **A** Diffuse BM infiltration by leukaemic cells. Dysplastic megakaryocytes are also present (arrow). **B** Mononucleated blasts and dysplastic megakaryocytes (arrow) express cytoplasmic NPM1 (APAAP immunostaining, x400). **C** Sanger sequencing of exon 11 showing a heterozygous 8 nucleotides insertion leading to a stop codon at amino acid 275. **D** Schematic representation of the new mutant protein (Mut) compared with the wild-type (WT). Analysis of the new protein sequence predicted a truncated protein (274 aa length), with a newly acquired NES motif (VxxxFxxLxIx). Both proteins are recognized by the anti-NPM1, Clone 376 mAb: monoclonal antibody.

A 64-year-old woman presented with fatigue, diarrhea, nausea, dyspnea and fever. CBC showed WBC 43.3 × 10^9^/L, Hb 3.5 g/dL and platelets 67 × 10^9^/L. The BM was diffusely infiltrated by leukaemic cells with myelomonocytic (FAB-M4) appearance expressing cytoplasmic NPM1 (Fig. 4A, B) with no mutations at *NPM1* exon 12; *FLT3* was wild-type and the BM karyotype was normal. She received a ‘7 + 3’ induction regimen, achieving CR. We then administered two consolidation cycles with intermediate dose cytarabine and daunorubicin. She is now in CR, 6 years after the initial diagnosis.

### Questions and recommendations

*NPM1* mutations almost exclusively affect exon 12 [68] and all of them cause changes at the C-terminus of NPM1 (mutation of tryptophans and creation of a NES motif) resulting in enhanced nuclear export of NPM1 mutants and their aberrant accumulation in the cytoplasm of AML cells [63]. Very rare mutations involving exons 9 [69], 11 [70] and 5 [71] also lead to cytoplasmic delocalization of mutant NPM1 through a similar molecular mechanism.

Cytoplasmic expression of NPM1 (a surrogate for *NPM1* mutations) can be detected by IHC in BM biopsies [3]. In our patient, the discrepancy between IHC (cytoplasmic NPM1) and conventional molecular analysis of exon 12 (absence of *NPM1* mutation) prompted us to study the entire *NPM1* coding sequence, revealing a mutation at exon 11 (Fig. 4C), similar to the one we previously described [70]. As expected, this mutation caused the loss of the two C-terminal tryptophans and the creation of a NES (Fig. 4D), explaining the abnormal cytoplasmic localization of NPM1.

IHC represents a valuable tool for predicting all *NPM1* mutations [72]. NGS can also identify all *NPM1* mutations but the commercially available panels are designed to recognize mutations at exon 12 only. Thus, they must be adapted for this purpose. Without IHC or NGS, cases like ours would be wrongly assigned to the ELN intermediate risk category (*NPM1* wild-type without *FLT3*-ITD) rather than to the favorable risk group (*NPM1*-mutated without *FLT3-*ITD). Our patient had good outcome after CHT alone, suggesting that cases with exon 11 mutations may behave similarly to those with exon 12 mutations, but confirmatory studies are needed. MRD monitoring of these very rare *NPM1* mutations may require designing a patient-specific RT-qPCR assay [73].

## Case 6: *NPM1*-mutated AML carrying *BCR-ABL1*

**Fig. 5:**
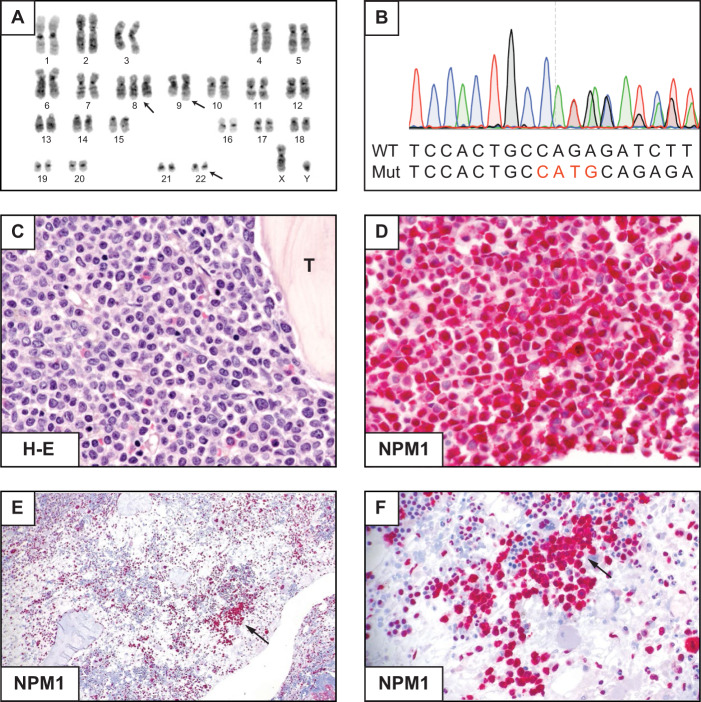
AML with *NPM1* mutation and *BCR-ABL1* rearrangement. **A** G-banding showing a subclone with the following karyotype: 47,XY,t(9;22)(q34;q11),+8 (see the text for the full karyotype). **B** Sanger trace of *NPM1* exon 12, demonstrating mutation B. **C** Massive BM infiltration by leukaemic cells. T indicates bone trabecula (BM biopsy, haematoxylin-eosin, x400). **D** Leukaemic cells exhibit aberrant cytoplasmic expression of NPM1 (BM biopsy, APAAP staining, x400). **E** BM re-evaluation after the first cycle of CHT. Good recovery of normal haematopoietic cells showing nucleus-restricted NPM1 positivity (indicative of *NPM1* wild-type). A small cluster of residual leukaemic cells with cytoplasmic NPM1 is seen (arrow) (BM biopsy, APAAP immunostaining, x100). **F** Higher magnification of the small cluster of NPM1 cytoplasmic leukaemic cells (arrow) shown in Fig. 4E (BM biopsy, APAAP immunostaining, x400).

A 49-year-old man presented with fever, myalgia, haematuria, WBC 98.7 × 10^9^/L, Hb 9.9 g/dL and platelets 14 × 10^9^/L. BM evaluation performed at another Institution was diagnostic of AML with 46,XY,t(9;22)(q34;q11)[9]/47,idem,+8[7]/46,XY[4]; a *BCR-ABL1* rearrangement (p210 fusion) (Fig. 5A) and *NPM1* mutation type B were detected (Fig. 5B), whilst *FLT3* was wild type. Dasatinib was started at a different institution but discontinued after 2 weeks due to pericardial effusion. He presented at our Institution with WBC 2.3 × 10^9^/L, Hb 7.8 g/dl and platelets 19 × 10^9^/L. BM biopsy was massively infiltrated by myeloid (FAB-M2) blasts (Fig. 5C) expressing cytoplasmic NPM1 (Fig. 5D), indicating no response to dasatinib. Sequencing *ABL1* exons 4–10 showed no mutations. A ‘7 + 3’ regimen induced partial response (10% residual NPM1 cytoplasmic-positive AML cells) (Fig. 5E, F). He was re-induced with fludarabine, cytarabine and idarubicin, achieving CR whilst fluorescent in situ hybridization (FISH) revealed 0.5% of nuclei with *BCR-ABL1*. He underwent allo-HSCT from an HLA-identical sibling and is now in molecular CR for *NPM1* and *BCR-ABL1*, 12 months after the allotransplant.

### Questions and recommendations

*NPM1* mutations have been reported in de novo AML with *BCR-ABL1* but not in chronic myeloid leukaemia in blastic phase [74–76]. How should this patient be classified? In the 2017 WHO classification [5], *NPM1*-mutated AML and AML with *BCR-ABL1* represent a ‘distinct’ and a ‘provisional’ entity, respectively [5]. Therefore, *NPM1* mutations supersede *BCR-ABL1* and our case should be diagnosed as *NPM1*-mutated AML, annotating the presence of *BCR-ABL1* [5]. Notably, leukaemic cells showed CD34 negativity that is more consistent with *NPM1*-mutated AML [3] than with *BCR-ABL1* AML [5]. The relative contribution of the two genetic abnormalities to leukaemogenesis remains to be established.

What is the prognostic impact of these genetic alterations? In the 2017 ELN model, AML with *BCR-ABL1* is regarded as high-risk disease [77] whilst *NPM1*-mutated AML without *FLT3*-ITD has a relatively good outcome [6]. ELN recommendations [6] do not comment on the combination of *NPM1* mutations and *BCR-ABL1*. Interestingly, two patients carrying *NPM1* mutation and *BCR-ABL1* were alive 36 and 71 months after diagnosis [74], suggesting that they may behave more like a *NPM1*-mutated AML than as de novo AML with *BCR-ABL1*. However, further studies are required.

### Practical recommendations for the clinical management of *NPM1*-mutated AML patient

*NPM1*-mutated AML should be suspected in a middle-age or older patient who presents with M4-M5 morphology [3], cup-like nuclei [78] or multilineage involvement [26] (case 1), a yet relatively preserved number of platelets despite high WBC count and negativity for CD34. Hyperleukocytosis usually associates with a concomitant mutation of *FLT3* or *RAS*. A low WBC count does not exclude *NPM1*-mutated AML since cases with *FLT3* wild-type may have this presentation, despite the BM tends to remain not hypocellular [79]. Independently of high WBC count, *NPM1* or/and *FLT3*-ITD mutations may associate with disseminated intravascular coagulation [80]. Extramedullary involvement may occur [7], especially in skin (case 2).

In haematological centers performing BM biopsy at presentation, demonstration by IHC of cytoplasmic NPM1 may serve as surrogate to molecular techniques in case of dry tap or myeloid sarcoma [7] (case 2). Moreover, as discussed in case 5, IHC could predict even rare *NPM1* mutations occurring at exons other than 12 and even *NPM1*-containg fusion transcripts [81].

Confirmation of diagnosis requires the identification of *NPM1* mutation by Sanger or the more sensitive NGS technique (detecting 1–5% of mutated cells, depending on the allele coverage). NGS can also identify concomitant driver mutations, even within subclones. Identifying the exact type of *NPM1* mutation [11, 82] is critical for setting-up the strategy of MRD monitoring (see below).

**Fig. 6:**
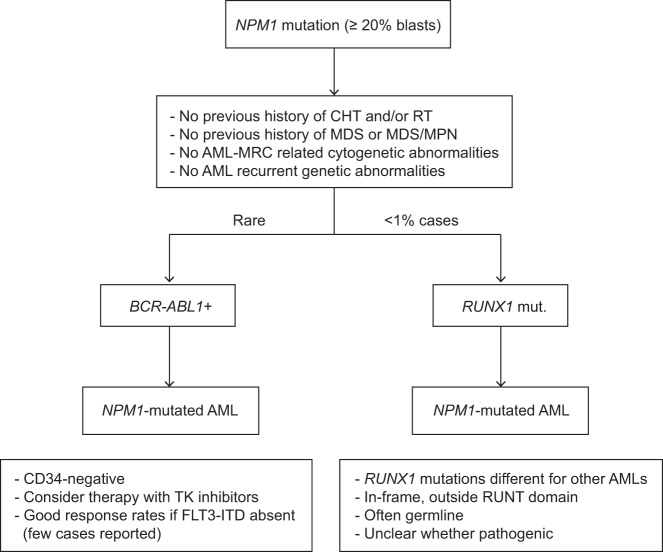
Differential diagnosis of NPM1-mutated AML. Decisional algorithm for distinguishing *NPM1*-mutated AML from AML with *BCR-ABL1* and AML with *RUNX1* mutations (two provisional entities of the 2017 WHO classification of haematopoietic tumors).

Once a *NPM1* mutation has been recognized, the definitive diagnosis of *NPM1*-mutated AML requires that other distinct and provisional AML entities of the 2017 WHO classification, such as AML-MRC (case 2), AML with *BCR-ABL1* (case 6, Fig. 6) and AML with *RUNX1* mutations (Fig. 6) are excluded. According to WHO 2017, the diagnosis of *NPM1*-mutated AML requires ≥20% BM blasts [5] (Fig. 6). However, the rare cases diagnosed as *NPM1*-mutated MDS show rapid transformation to AML [83] and respond better to CHT than to HMAs [84], suggesting that *NPM1* mutations may define AML irrespective of blast percentage [85]. Similarly, *NPM1*-mutated chronic myelomonocytic leukaemia (CMML) cases [86, 87] often carry a normal karyotype and tend to evolve rapidly to AML [86], especially those with high *NPM1* mutation allelic burden [87]. In our experience, they usually represent *NPM1*-mutated AML presenting with marked monocytosis.

Once the diagnosis of *NPM1*-mutated AML has been established, the patient should be assigned to one of the ELN categories, either favorable (if *FLT3* is wild-type or low ratio) or intermediate (*FLT3*-ITD^high^). Cases co-mutated for *NPM1* and *FLT3* should be possibly analyzed for *DNMT3A* to exclude the triple mutated genotype, which shows a particularly adverse outcome. As discussed in cases 1, 2, 3 and 4, risk stratification is important in guiding frontline (whether to use or not a FLT3 inhibitor) and post-remission therapeutic decisions (whether to perform or not allo-HSCT). Patient’s age and fitness are other important parameters to be considered for choosing between CHT and venetoclax-based regimens or for deciding whether to perform or not allo-HSCT.

**Fig. 7:**
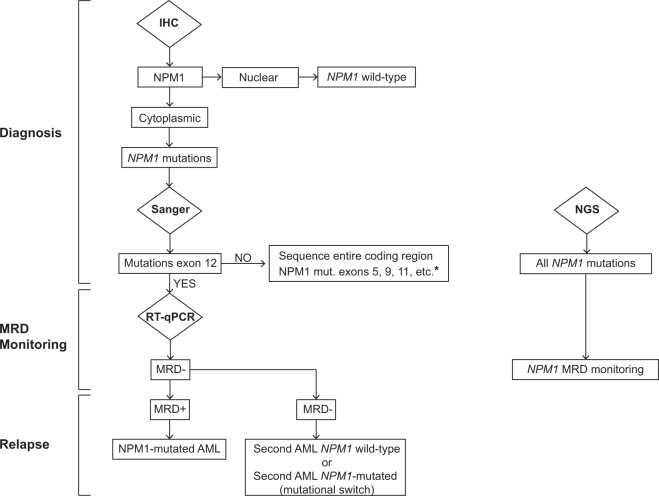
Decisional algorithm for the molecular diagnosis of *NPM1*-mutated AML and monitoring of MRD. Immunohistochemical analysis (IHC) on BM trephine discriminates between AML with nuclear localization of nuclephosmin (predictive of wild-type *NPM1* gene), and AML with cytoplasmic staining for NPM1 (predictive of *NPM1* mutations). Standard Sanger sequencing of *NPM1* exon 12 (involved in almost all cases) allows identification of the specific *NPM1* mutations. The discrepancy between IHC (cytoplasmic NPM1) and conventional molecular analysis of exon 12 (absence of *NPM1* mutation) should prompt to study the entire *NPM1* coding sequence to exclude mutations in other exons. Application of RT-qPCR to monitor MRD to be performed during CHT and at interval of 3 months for at least 2 years after the end of CHT + /− allo-HSCT. NGS has the potential to identify all *NPM1* mutations but the commercially available panels should be implemented to include, together with exon 12, at least exon 11, 9 and 5. *Should the entire coding sequence be wild-type, FISH to exclude very rare *NPM1* fusions should be considered. RNA sequencing should be also performed in these cases to identify novel *NPM1* translocations. NGS can be used also for MRD monitoring. Relapse with no detectable *NPM1* mutation points towards a diagnosis of second AML.

As discussed in cases 1 and 2, MRD monitoring (Fig. 7) during and after completion of CHT + /- allo-HSCT is a critical step in *NPM1*-mutated AML [24]. In fact, progressive raising of MRD levels is a reliable predictor of impending haematological relapse [15, 24, 88]. Patients in molecular relapse (as case 2) are candidates for pre-emptive therapy [31–33], although it still remains uncertain whether converting pre-transplant MRD positivity into negativity is clinically relevant. Relapse without detectable *NPM1* mutation points to a second AML [89–91] (Fig. 7).

## Conclusions and future perspectives

Although the presence of *NPM1* mutations cannot overcome the prognostic relevance of high-risk cytogenetic abnormalities (e.g. monosomy 7 defining AML-MRC), available data suggest that it probably supersedes the value of blast count. Indeed, the rare cases diagnosed as *NPM1*-mutated MDS or CMML often show features overlapping with AML [83–87]. Therefore, we envision that future issues of the WHO classification will identify *NPM1* mutations as genetic abnormalities sufficient to diagnose acute myeloid leukaemia regardless of the blast count, joining t(8;21), inv(16), t(16;16), and t(15;17).

Despite great progresses, improving standardization of MRD monitoring and therapy of *NPM1*-mutated AML remain a medical need. MRD monitoring is usually done by RT-qPCR [11]. However, highly sensitive NGS techniques for monitoring *NPM1* MRD have become available [92–94]. Digital droplet PCR may be an alternative, even for assessing rare *NPM1* mutations [95].

Incorporation of gemtuzumab ozogamicin has been advocated in the frontline treatment of *NPM1*-mutated AML [96–98]. Potential of venetoclax plus CHT [99–101] and mechanisms of resistance to venetoclax [102] should be further explored in *NPM1*-mutated AML. The good outcome of *NPM1*-mutated AML with *FLT3*-ITD^low^ was questioned in recent CHT-based studies [103–105], whilst clearly emerged in trials using CHT plus midostaurin [106]. Thus, the best post-remission therapy for this genotype remains controversial. Translation into clinic of XPO1 [107] and MLL-Menin inhibitors [27, 108], alone or combined with FLT3 inhibitors [109] is warranted. Finally, NPM1 mutant neoantigens complex may be a potential target for immunotherapy [110].
